# A new high-resolution 3-D quantitative method for analysing small morphological features: an example using a Cambrian trilobite

**DOI:** 10.1038/s41598-018-21088-4

**Published:** 2018-02-12

**Authors:** Jorge Esteve, Yuan-Long Zhao, Miguel Ángel Maté-González, Miguel Gómez-Heras, Jin Peng

**Affiliations:** 1 Departamento de Geociencias, Universidad de los Andes, Cra 1 No 18A-10, AA 4976 Bogotá D.C, Colombia; 2 Resources and Environmental Engineering College, Guizhou University, Guiyang, 550025 China; 3 Department of Cartography and Terrain Engineering, Polytechnic School of Ávila, University of Salamanca, 05003 Ávila, Spain; 4 C.A.I. Arqueometry and Archaeological Analysis, Complutense University of Madrid, 28040 Madrid, Spain; 5 Departamento de Geología y Geoquímica Universidad Autónoma de Madrid, 28049 Madrid, Spain; 6 Instituto de Geociencias (CSIC, UCM), 28040 Madrid, Spain

## Abstract

Taphonomic processes play an important role in the preservation of small morphological features such as granulation or pits. However, the assessment of these features may face the issue of the small size of the specimens and, sometimes, the destructiveness of these analyses, which makes impossible carrying them out in singular specimen, such as holotypes or lectotypes. This paper takes a new approach to analysing small-morphological features, by using an optical surface roughness (OSR) meter to create a high-resolution three-dimensional digital-elevation model (DEM). This non-destructive technique allows analysing quantitatively the DEM using geometric morphometric methods (GMM). We created a number of DEMs from three populations putatively belonging to the same species of trilobite (*Oryctocephalus indicus*) that present the same cranidial outline, but differ in the presence or absence of the second and third transglabellar furrows. Profile analysis of the DEMs demonstrate that all three populations show similar preservation variation in the glabellar furrows and lobes. The GMM shows that all populations exhibit the same range of variation. Differences in preservation are a consequence of different degrees of cementation and rates of dissolution. Fast cementation enhances the preservation of glabellar furrows and lobes, while fast dissolution hampers preservation of the same structures.

## Introduction

Preservation is an important factor that palaeontologists must deal with when studying fossils. This is a two-folded concept; firstly concerning with preservation in a taphonomic sense and secondly, concerning with the preservation of specimens when carrying out morphological studies. Vertebrates are paradigmatic fossils; taphonomic experiments can be carried out to test how decay affects their general morphology^1^, as well as processes in bone diagenesis^2^. Invertebrates have also been the subject of taphonomic experiments that can have phylogenetic implications^3^ and biostratinomic factors are often well known in these animals^4–6^. Based on this, it is becoming increasingly clear that small morphological features (e.g. small furrows or pits) of fossil invertebrates are useless or at least difficult to use for taxonomic purposes due to the high variability in their preservation^7^; nevertheless, in many cases, taxonomists still use these characters to differentiate species.

The hard external skeleton of trilobites exemplifies this difficulty. Recently, Esteve *et al*.^8^, using geometric morphometric methods, demonstrated that the cranidial morphology of *Oryctocephalus indicus*^9^ from South China and *Oryctocephalus* “*reticulatus*”^10^ from the Molodo River in Siberia is rather constant and that both species share the same morphospace. However, there is one morphological difference that remains: *O*. *indicus* (=*O. “reticulatus”*) from Siberia has one transglabellar furrow in most of the specimens, whereas *O*. *indicus* from South China exhibits more variation in the number of transglabellar furrows. Sundberg *et al*.^11^ suggested *O. reticulatus* might also be present in South China because some specimens from the Kaili Formation that were previously assigned to *O. indicus* possess only one transglabellar furrow. However, the co-occurrence of both taxa in the same strata in China seems very unlikely and variation in taphonomy could be a better explanation for this apparent morphological variation. Moreover, *Oryctocephalus indicus* from West USA typically has three transglabellar furrows in most specimens (Fred Sundberg personal communication, 2015), although some specimens with one or two transglabellar furrows have also been found^8^. There are two possible explanations for these differences. Firstly, even though geometric morphometrics suggests that both putative species are in fact the same species, it is known that different taxa can share the same cranidial morphospace^12^. Consequently, a different approach (e.g. outline-based) should be used to assess the results of the landmark-based approach employed by Esteve *et al*.^8^. The second possible explanation is that taphonomic bias is behind the obliteration of the second and third transglabellar furrows in the specimens from Siberia. However, if the second statement is true, how can we assess the variation of morphological traits smaller than 100 µm?

The second level of preservation, which is related to the conservation of singular specimens, such as those in museums and collections and specially type material, it is also a necessary concern, as preserving these specimens has a unique social and scientific importance. Hence, the use of non-destructive methodologies for morphological studies is, more often than not, a must. The aim of this work is to adopt a new high-resolution non-destructive method of 3-D reconstruction using an optical surface roughness meter to visualize small morphological structures around 50–100 µm in size (i.e. transglabellar furrows in the glabella) and thereby assess morphological differences in the second and third transglabellar furrows within and among three *O. indicus* populations (USA, Siberia and China).

## Results

### Optical surface roughness meter (OSRM)

**Figure 1:**
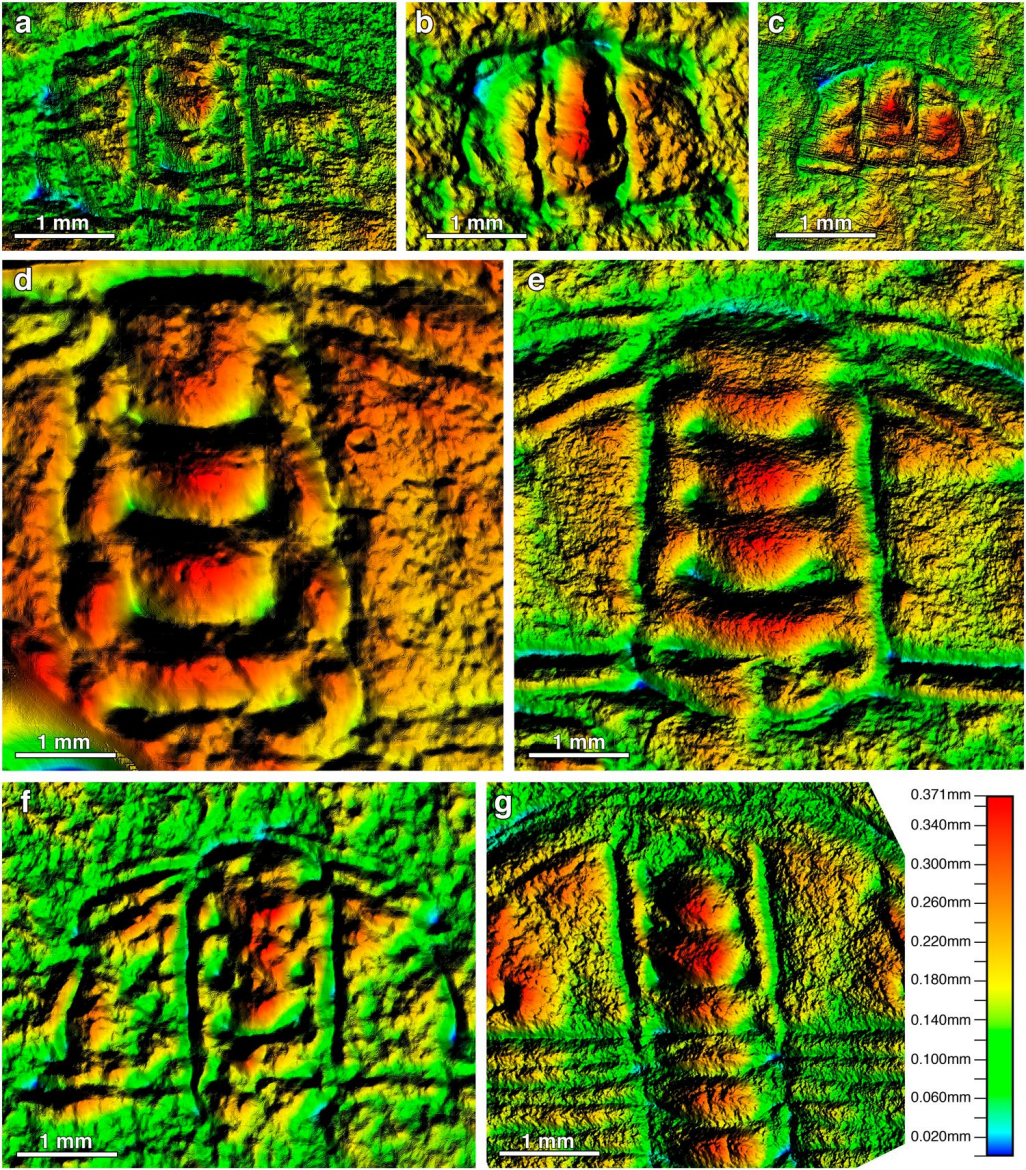
Digital elevation models (DEMs) (**a–c**) *Oryctocephalus indicus* (Reed, 1910) from Siberia (**a**) MPZ2017/448; (**b**) MPZ2017/449; (**c**) MPZ2017/450; (**d–e**) *Oryctocephalus indicus* (Reed, 1910) from Split Mountain, Nevada, Great Basin, USA d) MPZ2017/432; (**e**) MPZ2017/433; (**f–g**) *Oryctocephalus indicus* (Reed, 1910) from the Kaili Formation, South China f) MPZ2017/467; (**g**) MPZ2017/468.

**Figure 2:**
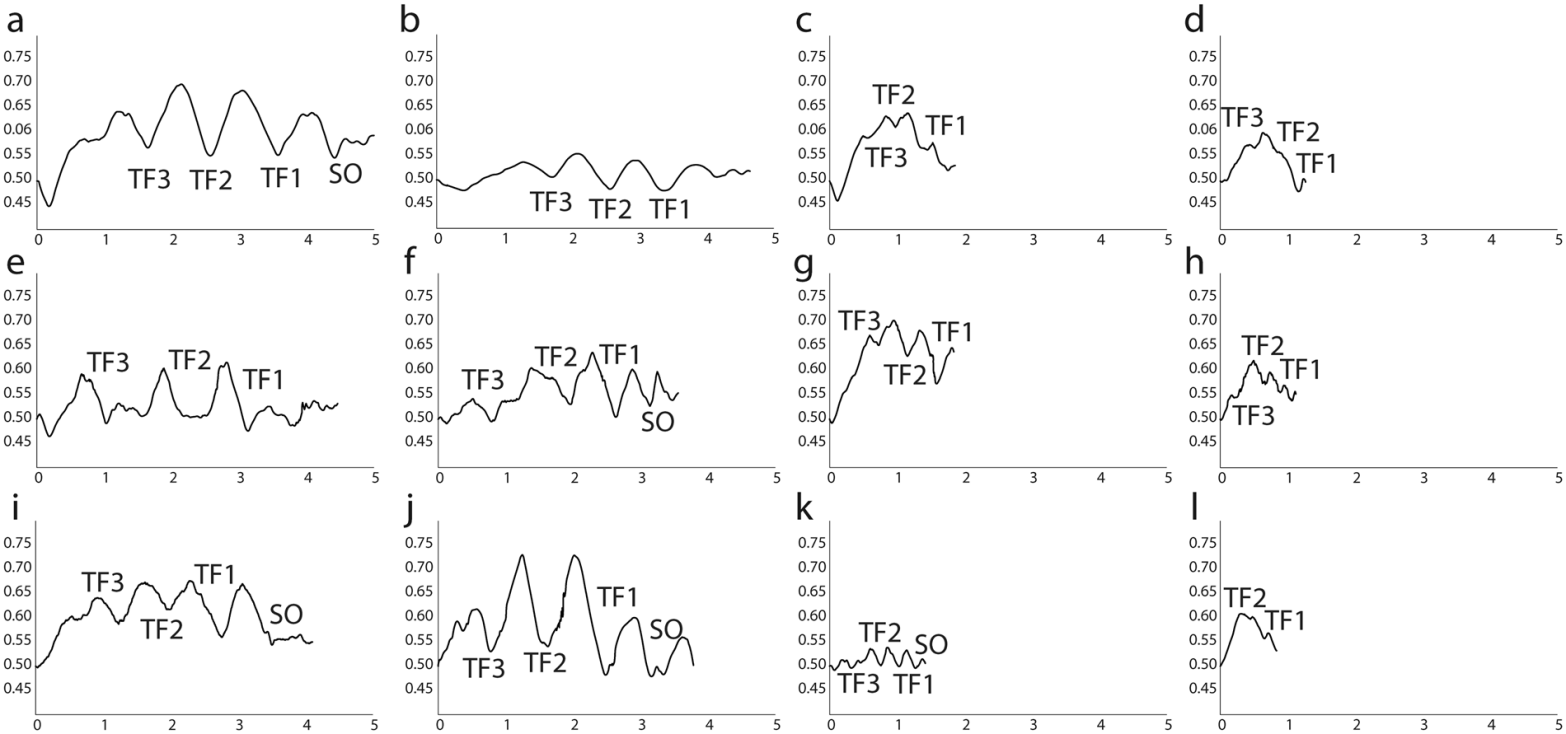
Profiles obtained with the DEM of a-d) Specimens from Split Mountain, Nevada, Great Basin, USA; (**a**) MPZ2017/434; (**b**) MPZ2017/435; (**c**) MPZ2017/436; (**d**) MPZ2017/436; (**e**–**h**) Specimens from Siberia; (**e**) MPZ2017/451; (**f**) MPZ2017/452; (**g**) MPZ2017/453; (**h**) MPZ2017/454; (**i**–**l**) Specimens from the Kaili Formation, South China; (**i**) MPZ2017/469; (**j**) MPZ2017/470; ki) MPZ2017/471; (**l**) MPZ2017/442. (Vertical and horizontal axis in mm).

The OSRM analysis produced digital elevation models (DEM, Fig. 1) in sagittal and exsagittal profiles of each specimen. These profiles allowed visualization of the preservation of the transglabellar furrows, even those with poor development, and quantification of the difference of elevation rate between the populations. Both profiles (i.e. sagittal and exsagittal) provided similar results; consequently, here we only report the axial profile, which is the main focus of the current study. The DEMs of each specimen of the three populations show roughly the same ranges of elevation (Fig. 2). Though the DEMs of both, largest (Fig. 2a,b) and the smallest (e.g. Fig. 2g,k) specimens show the same variation in the elevation range. Supplementary information provides data to visualise all DEMs. DEMs show that small specimens also have well preserved transglabellar furrows (Fig. 2h,k), though they are unclear in some cases (Fig. 2l). The DEMs show that transglabellar furrows are relatively closer together in smaller specimens, and move further away from one another through ontogeny. Synoptically, Fig. 2 shows the axial profile of 12 specimens (4 from each palaeocontinent) in which glabellar length ranges from *c*. 1.5 mm to *c*. 5 mm. The transglabellar furrows are visible in all specimens with a glabellar length of around 5 mm. However, while in the specimens from the USA the glabellar lobes are rather rounded, in the specimens from Siberia and China the glabellar lobes are sharped/pointed. The same feature can be observed in the deep part of the transglabellar furrows, which are more rounded in the American specimens and pointed in Chinese and Siberian specimens. These two types of preservation allow us to define two kinds of glabellar furrows and lobes according to their profile: i) Sharper and ii) Rounded. These two types of furrows and lobes seem to be closely related with the preservation, and suggest that *O. indicus* from USA was subject to cementation early in diagenesis, whereas in the specimens from China and Siberia cementation was delayed until later in diagenesis (see discussion below).

**Figure 3:**
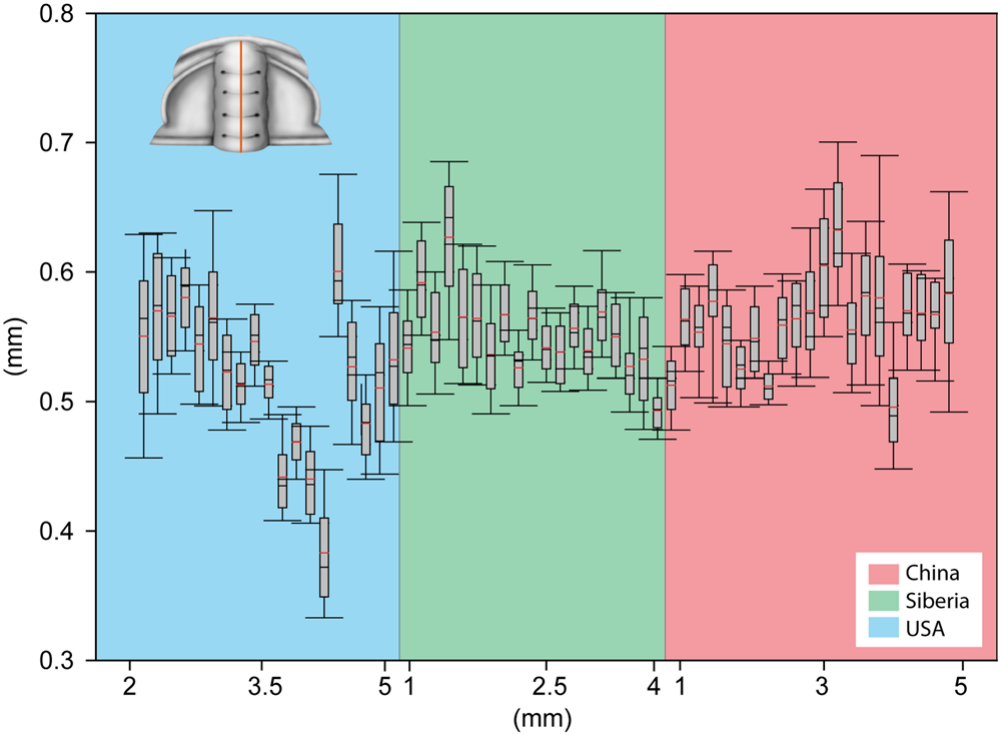
Boxplot showing the range variation in the axial profiles of the three analysed populations.

The morphological variation of the profiles described by the DEMs is here illustrated in a boxplot (Fig. 3). This demonstrates that the individuals from the USA and, especially, China exhibit considerable variation. In contrast, the DEMs show a much more narrow range of elevations in the Siberian population. This suggests that the Siberian sample is more homogeneous in terms of differences of elevation while the American and Chinese population present more variation. This difference in the elevation ranges is not size related, since the large and small specimens share the same elevation ranges (Fig. 3).

### Geometric morphometric analysis (GM)

**Figure 4:**
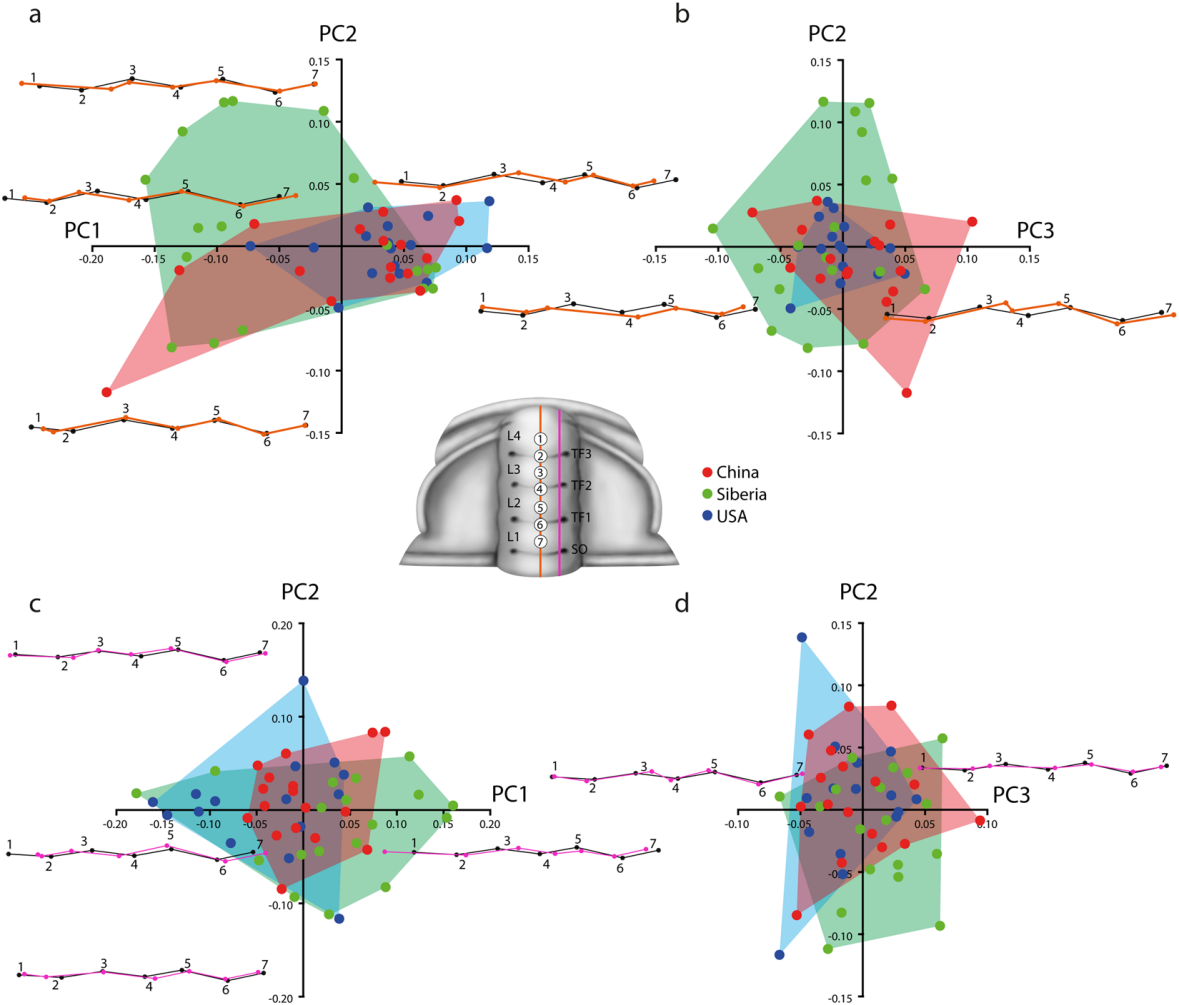
Principal Component Analysis (PCA) of mature *Oryctocephalus indicus* (Reed, 1910); percent variation summarized by each axis shown the text. The reconstruction shows the position of the seven landmaks. (**a,b**) Morphospace defined by the first three principal components of PCA of the sagittal profiles, showing the wireframe visualization of the profile variation along PC1, PC2 and PC3. (**c,d**) Morphospace defined by the first three principal components of PCA of the exsagittal profiles, showing the wireframe visualization of the profile variation along PC1, PC2 and PC3.

**Figure 5:**
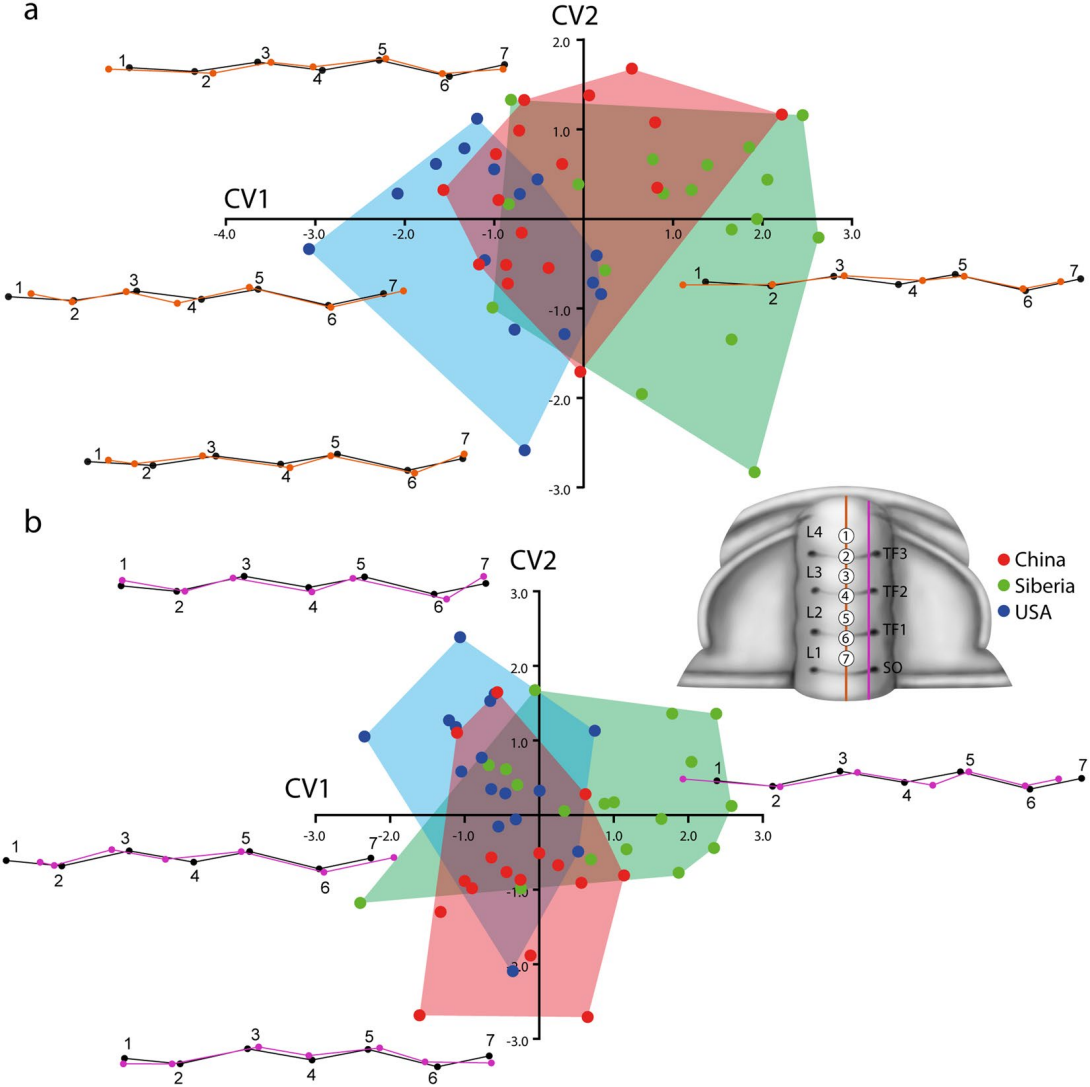
Canonical Variates Analysis (CVA) of mature *Oryctocephalus indicus* (Reed, 1910); percent variation summarized by each axis shown the text. The reconstruction shows the position of the seven landmaks (**a**) Morphospace defined by the first two canonical axis of the sagittal profiles, showing the wireframe visualization of the profile variation along CV1 and CV2. (**b**) Morphospace defined by the first two canonical axis of the exsagittal profiles, showing the wireframe visualization of the profile variation along CV1 and CV2.

Two different profiles have been analysed. Consequently, two GM analyses were performed, one for profile-1 (sagittal profile, Fig. 4a,b, Fig. 5a) and one for profile-2 (exsagittal profile, Fig. 4b,c, Fig. 5b).

Axial profile (orange, Figs 4 and 5): The Principal Component Analysis (PCA) of the axial profile shows overlap in the deepness of transglabellar furrow among the three populations analysed (Fig. 4a,b). The first three PCs account for 79% of the total shape variation providing a reasonable approximation of the total amount of shape variation across the transglabellar furrows. PC1 accounts for 50.8% of the total variation and relates primarily to the position of L1, L3 and L4 (landmarks 1, 3 and 7) and the position of the TF2 (landmark 4) to the rest of the glabellar profile. The position and depth of the TF3 is not affected in this axis. Negative scores in the PC1 correspond to the more anterior position of L1 and L4 (landmarks 1 and 7), while L2 and TF2 (landmarks 3 and 4) move towards rear position. This morphospace is occupied mainly by specimens from Siberia and a few from China. Positive scores in the PC1 correspond with forms with the L4 (landmark 1) in rear position closer to the TF3 (landmark 2) and L1 in a more rear position further away of TF1. Differences in the relative position of the rest of the landmarks are negligible. Positive scores in the PC1 are occupied mainly by specimens from USA and China. PC2 accounts for 17.2% of the total variation and is primarily related to the development of the TF3 (landmark 2), poorly developed and situated close to the L3 (landmark 3) in negative scores and better developed in positive scores in more anterior position close to the L4 (landmark 1). Positive values correspond with specimens from Siberia as well as negative values, while most of the specimens from USA and China are close to the mean profile shape. Relatively high amount of variation, 11.8%, is accounted for in the PC3. The variation in the PC3 relates mainly to the position of L2 and L3 (landmarks 3 and 5), which are closer in positive scores and closer to the mean shape in negative values. The depth of the TF3 (landmark 4) also varies from shallow forms in positive scores to deeper forms in negative scores. All populations encompass the scores in the PC3.

The Goodall’s F test (Procrustes ANOVA) shows significant similarities among populations (Centroid size: F = 6.58, df = 2, p < 0.003, shape: F = 2.88, df = 20, p < 0.0001). Pairwise comparison shows no significant differences between samples (Table 1). The axial profile of the specimens from Siberia compared to those from the USA are the most different of the sample, however the F-value is still significant lower. The CV analysis (Fig. 5a) displays significant similarities among the three populations examined (10000 permutation runs). The Mahalanobis and Procrustes distances between samples are very small, suggesting that the shape of TFs and glabellar lobes in the sagittal profiles in all three samples are very similar in shape. (Table 2). While positive values in the CV1 are occupied mainly by specimens from Siberia with the L4 (landmark 1) in an anterior position compared with the mean profile shape, negative scores in the CV1 are occupied by the tree populations with only American specimens in higher negative values. The variation in the negative CV1 values relates to the position of TF2 (landmark 4), which is slightly situated in a rear position and the L4 (landmark 1), which is slightly situated in a rear position.

**Table 1:** Goodall’s F-test among groups corresponding to the sagittal profiles (SP) and exsagittal profiles (EP).

Profiles comparison	Goodall’s F-test	
	F	*P*
China SP to Siberia SP	2.38	0.0098
China SP to USA SP	1.58	0.110
Siberia SP to USA SP	5.17	<0.0001
China EP to Siberia EP	2.24	0.0157
China EP to USA EP	0.75	0.6733
Siberia EP to USA EP	4.26	<0.0001

**Table 2:** Mahalanobis and Procrustes distances with P-values from permutation tests (10000 permutation rounds) for Mahalanobis and Procrustes distances among groups corresponding to the sagittal profiles.

	Mahalanobis distance				Procrustes distance			
	China		Siberia		China		Siberia	
	R	*P*	R	*P*	R	*P*	R	*P*
Siberia	1.4501	0.0756			0.0627	0.0628		
USA	1.4244	0.0619	1.4958	0.0258	0.0417	0.1733	0.0814	0.0036

Exsagittal profile (purple, Figs 4 and 5): The Principal Component Analysis (PCA) of the exsagittal profile shows overlap in the amount of the transglabellar furrow deepness among the three populations analysed (Fig. 4c,d). The first three PCs account for 80% of the total shape variation providing a reasonable approximation of the total amount of shape variation across the transglabellar furrows. PC1 accounts for 48.3% of the total variation and relates primarily to the position of L4 (landmark 1) to the rest of the glabellar profile, while the depth of the TF3 is not affected in this axis. Negative scores in the PC1 correspond with L4 closer to the TF3 and positive scores correspond with L4 in a most anterior position. The relative position of the rest of the landmarks does not vary appreciably. PC2 accounts for 21.7% of the total variation and is primarily related to the development of the TF3 (landmark 2), which is not developed in negative scores and poorly developed in positive scores. A relatively high proportion of the variation between specimens is controlled by the position of the L3 and TF2 (landmarks 3 and 4) (PC3, 11.6% of total variation). The two glabellar lobes show antagonist behaviour; positive scores have specimens with both lobes very separate and negative scores have specimens with lobes relatively closer.

The Goodall’s F test (Procrustes ANOVA) shows significant similarities between populations (Centroid size: F = 5.53, df = 2, p < 0.007, shape: F = 3.06, df = 20, p < 0.0001). Pairwise comparison shows no significant differences between samples (Table 1). As for the sagittal profile, the exsagittal profile of *O. indicus* from Siberia compared to those from the USA are the most different of the sample, however the F-value still significant lower. The CV analysis (Fig. 5b) displays significant similarities among the three populations examined (10000 permutation runs) The Mahalanobis and Procrustes distances between samples are very small, suggesting that the shape of TFs and glabellar lobes in the exsagittal profiles in all samples are very similar in shape (Table 3). Positive values in the CV1 are occupied mainly by specimens from Siberia with the L3 (landmark 1) in a very anterior position and the TF2 in a posterior position compared to the mean profile shape. Few specimens from Siberia and USA take place in the negative values of the CV1. Positive scores in the CV1 are occupied mainly by American specimens with the TF1 (landmark 6) and TF2 (landmark 6) slightly deeper than the mean shape. Chinese samples takes place in negative values of the CV2 and differ from the American population in having shallower transglabellar furrows.

**Table 3:** Mahalanobis and Procrustes distances with P-values from permutation tests (10000 permutation rounds) for Mahalanobis and Procrustes distances among groups corresponding to the exsagittal profiles.

	Mahalanobis distance				Procrustes distance			
	China		Siberia		China		Siberia	
	R	*P*	R	*P*	R	*P*	R	*P*
Siberia	1.3790	0.0745			0.0506	0.0740		
USA	0.8743	0.8036	1.9611	0.0005	0.0395	0.2361	0.0836	0.0084

## Discussion and Conclusions

The optical surface roughness meter (OSRM) is a powerful tool that can be used to generate digital elevation models (DEMs) for assessing small morphological features (<0.1 µm). This new technique allows us to better understand the effects of taphonomy on the transglabellar furrows (TFs) of *Oryctocephalus indicus*. Thanks to the OSRM, we were able to assess the variation in very small morphological features by means of a geometric morphometric approach. The DEMs allowed a 3-D quantification of these morphological traits and comparison between populations.

**Figure 6:**
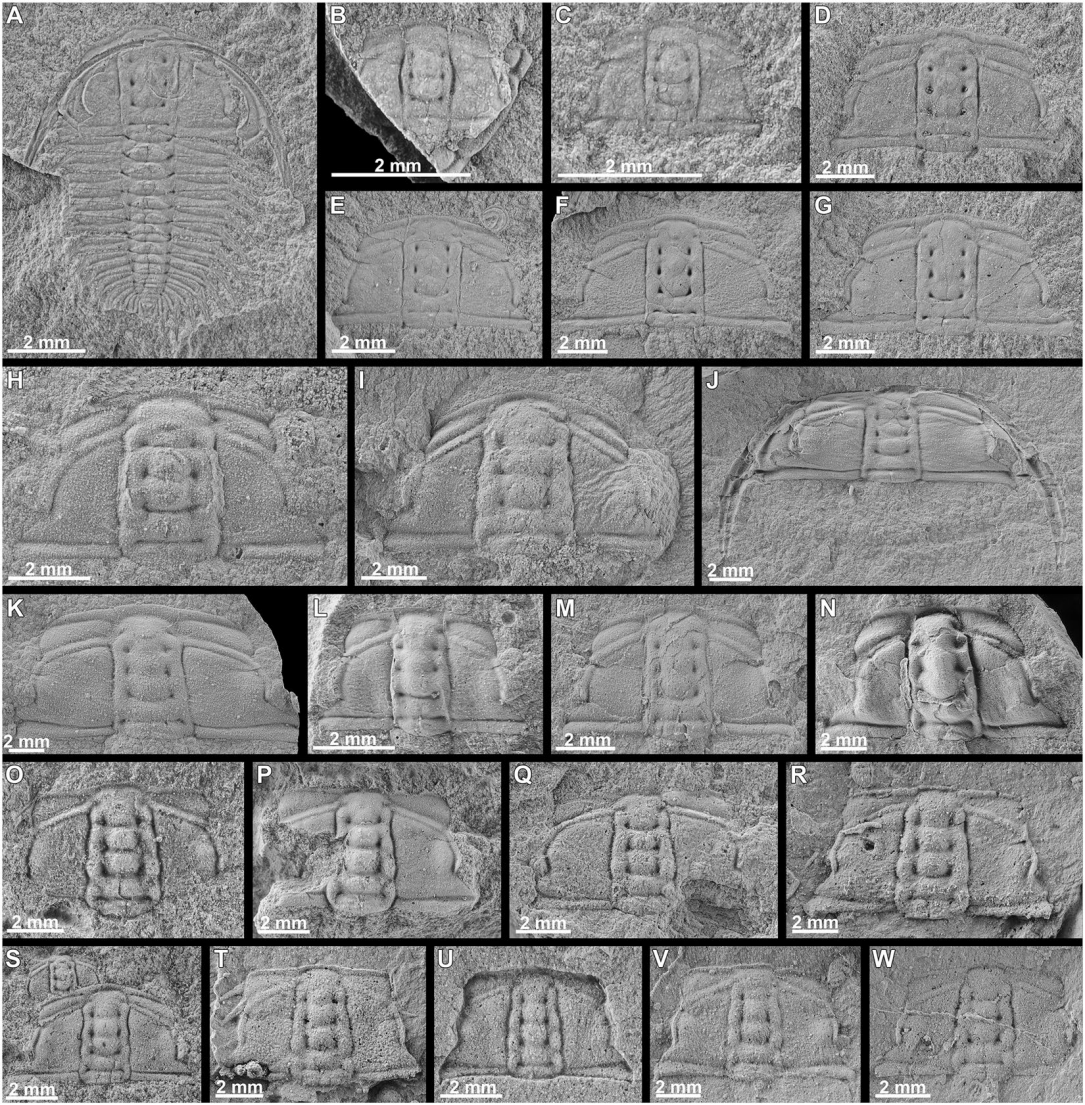
*Oryctocephalus indicus* (Reed, 1910). (**A–G**); MPZ2017/448—MPZ2017/454 from Siberia; (**H–N**); MPZ2017/475—MPZ2017/481 from the Guizhou Province, South China South China and (**O-W**); MPZ2017/434—MPZ2017/442 from the Split Mountain, Nevada, Great Basin, USA.

All studied sections bear specimens typically preserved as high-quality internal and external molds, preserving in many cases fine details of exoskeletal prosopon (Fig. 6). This is especially true (42% of the external moulds preserve remains of ornamentation, n = 72) in the external molds from Siberia (Fig. 6A–G). By contrast, there are fewer specimens preserving fine details in the material from China (<5% of the specimens show ornamentation, n = 554, Fig. 6H–N) and very few examples of this in the material from the USA (<2% of the specimens show remains of ornamentation, n = 331, Fig. 6O–W). In addition, in China is very common to find specimens preserved through mineral replacement as calcite; during weathering, the original cuticle was dissolved, and sometime later calcite was precipitated in the voids. The brown parts in fossils arise from where the calcite was stained black by Fe-oxides (from weathering of pyrite in the rocks) and Mn. The preservation mode is closely related to how the dissolution of the original exoskeleton occurred, which could happen following compaction of the sediments during burial or after burial. It is important to know how dissolution occurred (during or after burial) because this establishes new features in the architecture and general morphology of the cephalon. The OSRM analysis has been especially useful to assess the effects of dissolution. While specimens with a low degree of dissolution present glabelar lobes and furrows that are well defined and rounded (e.g. *O. indicus* from USA), the specimens that suffered more dissolution generally show sharper glabellar lobes and furrows (e.g. *O. indicus* from Siberia and China). This result agrees with an early cementation in diagenesis for *O. indicus* from the USA, which is supported by the wide spacing and lack of compaction of the cranidia and the lack of fine ornamentation. In addition, the presence of compaction-related fractures fits with this observation. Specimens from Siberia often present fractures which suggest that dissolution of the original exoskeleton must have occurred following compaction of the sediments during burial (Fig. 6A,E,F). This can greatly impact the preservation of small morphological features such as the TFs, which are often obliterated in the population from Siberia. However, the OSRM analysis shows that even some of those obliterated furrows can be visualized using new technologies, and the DEMs of specimens with obliterated furrows enable their analysis.

Geometric morphometric (GM) analysis quantifies the shape and variation of morphological traits. GM analysis demonstrates that an overlap of all shapes exists, but there is a trend towards effaced TFs in *O. indicus* from Siberia. The available data and the results suggest that the obliteration or effacement of the TFs could be as consequence of an early dissolution and diagenetic cementation of fossils. However, with regard to *O. indicus* from China, most of the specimens show a compaction-related plastic deformation and this suggests that the dissolution of the original exoskeleton must have occurred following compaction after burial (Fig. 6H–N). Esteve^13^ demonstrated that preservation of glabellar furrows does not depend on the lithology, thus specimens preserved in the same bed can show different degrees of preservation. PCA shows how the deepest of the TFs varies from obliterated TFs typical of the Siberian population to well-marked TFs in the American population, but overlap of both morphologies is seen in all populations. However, CVA shows a clear separation between *O. indicus* from Siberia with obliterated TFs and *O. indicus* from USA with well-marked TFs. These populations are linked by the Chinese population, which presents taphonomic features of specimens from USA (i.e. well-marked transglabellar furrows) and Siberia (i.e. obliterate transglabellar furrows). Although, *O. indicus* from USA and China show small Mahalanobis and Procrustes distances and low F-values, some of the tests are not statistically significant as a consequence of a small sample size and the extremely different shape of the furrows and lobes in few specimens. If only samples from the USA and China were analysed, it could be concluded that those samples represented different groups diagnosable by a unique range of continuous characters due to the absence of significantly different means and a minimal overlap. However, the sample of *O. indicus* from China overlap with USA and Siberia and cannot be distinguished from one another. Therefore, the authors do not believe these results warrant on themselves the separation of these groups into different species given the substantial overlap of samples in morphospace obtained thanks to the new 3-D quantitative method.

## Material and Methods

### Fossil sample

All the material used in this study is housed in the Paleontological Collections of the College of Resources and Environmental Engineering, Guizhou University, China (GTB, M) or at the Museo de Ciencias Naturales, University of Zaragoza (MPZ).

Guizhou Province, South China: Specimens come from the Wuliu-Zengjiayan section at Balang Village, Jianhe County, in the Miaoling Mountains, eastern Guizhou, China, which is one of the candidates to obtain the GSSP for the base of the Miaolingian Series and the Wuliuan Stage (former Cambrian Series 3, Stage 5 for geological details of this section, see^8,14–16^). 554 specimens from a short stratigraphic range (from a *c*. 10 m interval in the beds 14–15, see^14^) were photographed and used in this study, housed in the Paleontological Collections of the College of Resources and Environmental Engineering, Guizhou University, China, but 19 specimens (from a single population from the bed 14) that were scanned to create a digital elevation model are housed at the Museo de Paleontologia de la Universidad de Zaragoza (MPZ2017/467–485).

Molodo Section, Siberia, Russia: 72 cranidia of *O. “reticulatus”* were collected from a 10 cm interval in the Member IV slightly above the middle part of the Kuonamaka Formation. For more geological details of this section see^8,17^. 18 specimens from a single level Siberia were scanned to create a digital elevation model (MPZ2017/448–466).

Split Mountain, Nevada, Great Basin, USA: The Split Mountain section is the second candidate to obtain the GSSP for the base of the Miaolingian Series and the Wuliuan Stage (local Lincolnian Series and Delamaran Stage, former Cambrian Series 3, Stage 5 for geological details of this section, see^18,19^). 331 specimens to support the present study. These specimens were collected from two beds with different lithologies. The first bed corresponds with mudstones and the second with fissile shales. These specimens are housed at the Museo de Ciencias Naturales of the University of Zaragoza (MPZ). 16 specimens from a single population preserved in mudstones were scanned to create a digital elevation model (MPZ2017/432–447).

### Optical surface roughness meter

The need to make accurate and repeatable measurements of surface roughness is important in serval branches of geomorphology^20,21^, and the optical surface roughness meter is commonly used for this purpose in studies of the conservation of archaeological sites^22,23^. However, this is the first time the method has been applied to fossils. Select specimens were scanned with an optical surface roughness meter (TRACEiT, Innowep GMBH). This instrument measures the surface topography of a 5 × 5 mm area by acquiring three images angled at 120° apart with a resolution of 1500 lines in both x and y directions. From these images, the built-in software creates a 3D point cloud of the surface with a maximum accuracy of 1.5 μm, which is below the size of the structures to be analysed. Because of the limitations of the area covered by the equipment, measurements were focused on the glabella in large specimens, but smaller specimens fit fully within the measurable area. In addition to graphical information, the equipment output includes a *.MAP file, which corresponds to the 3D point cloud in text format in Cartesian coordinates. This file needed to be transformed into an absolute coordinates format so it could be opened with a 3D point cloud processing software, and custom software was used for this purpose. After this step, the file was imported into Global Mapper software to generate a digital elevation model (DEM) and subsequently obtain the profiles to be analysed (Figs 1, 2, 3). The DEMs are available under from the corresponding author on reasonable request, the dataset of each axial profile generated during the current study is available in this publication (Supplementary data 1).

### Shape analysis of the glabellar furrows and lobes

Geometric morphometric analyses were performed using an image of the profiles obtained with the optical surface roughness meter. Seven landmarks were chosen: landmarks 1, 3, 5 and 7 for glabellar lobes 4, 3, 2 and 1; and landmarks 2, 4 and 6 for transglabellar furrows 3, 2 and 1 (Figs 4–5). X–Y coordinates were obtained for all landmarks and the shape information was extracted using a full Procrustes fit, in order to standardize size, orientation and the position of each specimen. The morphological variation from different levels and from the whole section was analysed and synoptically viewed using principal component analysis (PCA) based on the covariance matrix. A Goodall’s F-test of Procrustes coordinates^24^ or Procustes ANOVA^25^ was used to test the significant morphological differences between samples^25–27^. Goodall’s F-test is a statistical approach that partitions variance of Procrustes distance rather than of landmark coordinates. Goodall’s F-test is the ratio of explained (between-group) to unexplained (within-group) variation in these distances. A bootstrapped F-test is more appropriate because it does not assume any isotropic normal distribution of landmarks around the mean; which is very unrealistic in biological or palaeontological samples. Thus, this test summarized the proportion of the shape variation that is not predicted by size. This test allows smaller sample size what other statistical test like MANOVA does not allow, and it is especially useful in geometric morphometric with small sample, where the number of free variables exceeding the number of degrees of freedom^27^. Therefore, when the F-value is very small it means that the variation between groups is negligible. The degree of variation in each sample was measured as within-group variance in Procrustes distance away from the group mean. The range of F-values obtained by randomly assigning specimens to populations is used to assess the probability that the observed F-value could be due to a random subdivision of an underlying single population. In order to test the difference of all multivariate samples, we also carried out a canonical variates analysis (CVA). CVA provides a scatter plot of specimens along the two first canonical axes, producing maximal and second to maximal separation between all groups (multigroup discriminant analysis^28^). In addition, we have calculated the Mahalanobis and Procrustes distances. The Mahalanobis distances in the transformed space measure the differences between groups relative to the within-group variation, and reflect the degree of separation between two groups relative to the within-group variation. Differences between pairs of shapes or deviations of individual shapes from the population average can be characterized by their magnitude, measured as a Procrustes distance, and their direction in the tangent space^29^. These transformations are a central idea for computing the canonical variate analysis^29^.

## Electronic supplementary material

Dataset 1
